# Health care overbooking cost minimization model

**DOI:** 10.1016/j.heliyon.2023.e18753

**Published:** 2023-07-27

**Authors:** Abdulaziz T. Almaktoom

**Affiliations:** Department of Operations and Supply Chain Management, Effat University, PO Box 34689, Jeddah, 21478, Kingdom of Saudi Arabia

**Keywords:** Healthcare, Scheduling system, Overbooking model, No-show cost

## Abstract

Challenges in the health care industry have confounded the provision of quality services for patients. Among many relevant concerns, the drive for cost-effectiveness and efficient care have placed considerable pressure on public health care systems and insurance coverage, amid existing barriers to restructuring entrenched systems. This study closely examines the factors impacting disruptions in health care scheduling systems using a structured case study in the health care industry. The study introduces a novel model to identify optimal overbooking capacity and minimize no-show costs. To address the complexity of this issue on a smaller scale, the model is implemented using a private hospital clinical platform in Jeddah, Saudi Arabia, instituting an overbooking reservation and queuing system in 14 departments to investigate the factors that influence disruptions and inefficacy of service provision, while also introducing a strategy for covering costs. The results identify the maximum amount of overbooking that can be made for each clinic. The cost-saving plan developed is expected to save each clinic a considerable sum, as opposed to randomly overbooking without any cost assumptions. Overall, if the clinics studied implemented this strategy, a total loss of no more than SAR. 2, 408 would be incurred from overbooking, in contrast to the exponentially growing amount of SAR. 10,000 that is currently lost on scheduling errors per year. The loss model developed has practical application as a tool for decision-making that includes no-show cost minimization variables.

## Highlights

• This study constructs and tests a viable decision tool for determining expected no-show costs in outpatient clinics.
• This study introduces a novel model for estimating suitable overbooking strategies to minimize loss.
• The model is tested using clinical platform data from 14 departments in a private hospital in Jeddah, Saudi Arabia, revealing that a total loss of no more than SAR. 2408 would be incurred from optimal overbooking, in contrast to the exponentially growing amount of SAR. 10,000 that is currently lost.
• The study presents and demonstrates the application of a valid tool for use in health care settings and numerous relevant industrial contexts that feature seats, reservations, and appointments and confront no-show costs, as well as offering an approach for integrating interactions with other relevant trends in future research.

## 1 Introduction

In the service operations sector, the practice of overbooking for cost efficiency is extremely common. Overbooking is primarily implemented in hotel and airline industries; however, it could also be considered for the health care industry, as it has wide potential for cost savings. Overbooking is a flexible scheduling technique with foundations in revenue management and offers considerable possibilities for lowering costs. For some industries, overbooking is considered to be a protective system in which reservations are accepted above capacity limitations to avoid the problem of no-shows or last-minute cancellations. As much as this concept may seem relatively effortless, it has been argued that overbooking carries its own set of complications in some scenarios. An example is in rental service frameworks, wherein an overbooking model could be far more complex in comparison to the airline industry, where different variables must be considered, including a variety of products, pricing, costs, and upgrading costs [1]. Overbooking models are constructed to predict expected loss in correlation with the number of seats overbooked, determining how much could be saved and related strategies. This concept is expansive and is widely applied in various industries but was primarily developed to serve the airline industry. LaGanga and Lawrence [2] proposed a model to mitigate the number of no-shows and increase productivity in health care clinics, demonstrating that even with inconsistent patient visit durations, the model was exceptionally profitable and cost effective to implement this model. Other studies have found that the expected number and ratio of no-shows to frequency is considerably high. According to Shabbir et al. [3], average no-shows at outpatient clinics in the UK are 12%, of which 30% claimed forgetfulness and 8% no longer felt the need for treatment. The authors also considered certain measures to lower the rate of forgetful no-shows, including telephone reminders prior to the appointment; however, instituting a high cancellation fee did not appear to make as much of a difference or added value as initially expected. Another set of variables that were found to be influential on the number of no-shows varied from providing a more comprehensive patient assessment, minimizing patient waiting time, maximizing flexible worker overtime, and maximizing health care provider productivity [3]. The main objective of this study is to investigate the factors that disrupt an efficient scheduling system and address them in a case study application in the health care industry. This study presents the case of a private local health care facility based in Jeddah, Saudi Arabia that has been operating for over six decades that has been acquired and run by a major health care firm in the past five years. It has 14 outpatient clinics that must develop a plan to minimize costs by monitoring and predicting the frequency of no-shows to enable the development of an effective overbooking scheduling solution.

The remainder of the study is organized as follows. Section 2 presents a review of the literature that provides an overview of traditional appointment scheduling methods, appointment scheduling and overbooking systems, the theoretical basis of the study, and details regarding the health care sector in Saudi Arabia. Section 3 provides the methodology, including the development of the loss model. Section 4 presents the case study and model implementation, and Section 5 introduces the research results and discussion. Finally, Section 6 presents the conclusion.

## 2 Literature review

### 2.1 Queuing system in health care clinical facilities

Hospital outpatient departments are challenged to provide quality health care services. They function as profitable units in hospital operations that invest in new technologies and seek to mitigate the cost of inpatient services. Despite this, many hospitals lack the required capabilities to manage and run outpatient departments, failing to address complaints of the long waiting times that result from long queues. This leads to overall patient dissatisfaction that must be managed to meet expectations by applying best practices and improved processes, particularly in developing countries [4].

Establishing a basic and clear queuing protocol could provide better insight and direction for investigating the outpatient clinic case study and determining the fixed numbers selected for clinic capacity. Clinics have a very simplified queuing model that is flexible and anticipatory regarding changes. Effective queuing and scheduling are two considerations that coincide with improved operational structure when applied [5]. Health care services rely heavily on perfecting and harmonizing the relationship between queuing and scheduling. The decision problem of an appointment time regards providing the patient with the appropriate time to receive care service according to the schedule, which optimizes the performance criteria [6]. According to Agrawal et al. [7], in the traditional appointment scheduling method, the clinic assigns a fixed day and time for a patient to be examined by a clinician. This method is used to optimize clinics’ operational objectives, although the approach lacks flexibility, resulting in patient dissatisfaction, increased no-show rates, and decreased revenue. In contrast, scheduling patients’ appointments to match their preferences benefits clinics; hence, matching patients with preferred physicians and appointment times can raise clinics’ revenue because it lowers no-show rates.

The panel size of a hospital indicates the number of potential patients for whom the hospital can provide the services. Not all of the potential patients or the concerned population demand health care services at a specific period, which could be a day, week, or month, which make a panel size larger than its service capacity. Accordingly, the panel size should accurately gage the number of potential patients to ensure profitability. In addition, both the panel size, and the number of potential patients should not be excessive to avoid long queues, long waiting times, and no-shows [6].

**Fig. 1:**
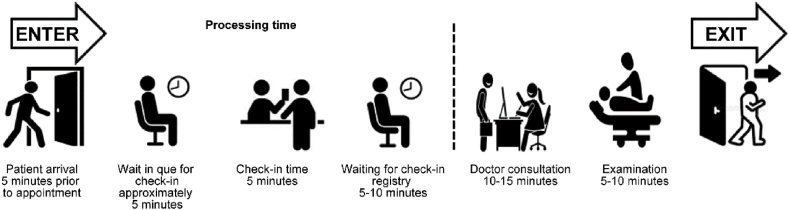
Ideal traditional queuing model for clinic services in the healthcare industry.

Fig. 1 illustrates the time duration and mandatory steps in an ideal clinic system, presenting two sides in a clinic queuing system; the first side, from arrival up to check-in, is a systematic aspect with a sense of structure and routine to a patient intake list. The second side is the dispositional aspect wherein the timeframe may vary depending on diagnosis and doctor consultation advice. The queuing layout suggested in the image first expects patients to arrive 5 min prior to the scheduled appointment time. The patient then takes a number for the check-in queue. Check-in waiting time takes from five to 10 min maximum, and once a patient is called, the check-in process takes approximately 5 min, covering a checklist of points, such as insurance coverage, patient history, and established payment. The next step transitions to the procedural side of the queuing system that includes examination and physician’s treatment recommendations. The entire process is approximated to optimally take 30 min. Such patient flow is seriously considered when structuring an organized scheduling system, as it provides an anticipatory analytical concept, but it lacks a clear perception on ratio of no-shows and the capacity of clinics’ operational capabilities.

### 2.2 Queuing theory

Queuing theory is considered a scientific approach for decreasing system inefficiencies and enhancing patient satisfaction. The theory has been applied to waiting line processes, analyses, and modeling to optimize the use of fixed resources according to predicted changing conditions of demand. Accordingly, operations managers at hospitals must understand the business process flow to be able to assess the operational performance of outpatient units for improvement by reducing waiting times [4]. According to Mehandiratta [8], the increasing cost of health care services can be attributed to inefficiencies in health care systems that can be reduced by applying queuing theory, given its effectiveness in cost reduction through decreasing delays and inefficiencies. In such systems, patients reserve an appointment with the outpatient unit, but face long waiting times before the actual physician consultation. Therefore, a scheduling system presents a tradeoff between patient waiting time and clinic overtime. A queuing system could be applied to reduce delays by synchronizing work between stages of service, including tests, discharge processes, and treatments. Another approach is scheduling resources (mainly physicians and nurses) to match patients’ scheduled arrivals in outpatient units. A third method is applying continuous system monitoring by regularly tracking and assessing patients’ wait times, locations, and diagnostic groups [8].

### 2.3 Appointment scheduling and overbooking system

The outpatient appointment system has an appointment scheduling window that signifies how many appointments can be scheduled in the future, which impacts the rate of no-shows and system efficiency. A reduction in the scheduling window results in an indirect reduction of waiting time, subsequently reducing no-show rates. This process generates more efficient use of the outpatient unit’s capacity. In contrast, rigid restrictions in a scheduling window can reduce the number of patients who actually show up for appointments, resulting in decreased revenue. Accordingly, hospital networks must develop optimal policies for booking systems. Clinics can schedule a number of patients that exceed their capacities to decrease the negative impact of no-shows, enhance patients’ access to services, and raise profits. This policy refers to application of the overbooking and cost minimization models that are commonly used in the travel industry. In the event that the overbooking level is not efficiently identified, it could result in a longer queue of patients and longer waiting times [6].

Moore and Wilson-Witherspoon, as cited in Al-Aomar and Awad [9], reported that hospitals lose about 3%–14% of potential revenue due to no-shows in the United States. This resulted in the development of multiple overbooking strategies to maintain revenue streams and ensure cost reduction in relation to no-shows. Moreover, long waiting times cause anger among patients around the clinic that can increase future no-shows. Parizi and Ghate [10] argued that scheduling issues affect health care operations; therefore, a clinic with one or more physicians must schedule dynamic appointment times for patients and prepare for no-shows and cancellations, considering that no-show rates vary widely in the literature depending on several factors. LaGanga and Lawrence [2] demonstrated large variations in no-show rates across geographic regions and among populations between 10% and 30% in hospitals and medical centers. Despite the prevalence of these issues across a wide range of applications, minimal research has been conducted regarding potentially applicable mathematical models and dynamic stochastic optimization, and there is relatively little research examining applications of patient categorization to scheduling problems that include dynamic and stochastically arriving patient requests [10].

### 2.4 Approaches to manage no-shows

The development of a no-show model that can accurately record the behavior of various patients scheduled to visit a clinic is the initial step in designing an overbooking system. The no-show rate may vary each day, and every patient could be assigned a high or low no-show rate. Accordingly, an outpatient clinic scheduling model that improves the prediction of no-show probabilities will be beneficial for the inclusion of no-show heterogeneity in overbooking considerations [11]. There are two major approaches to managing no-shows in health care facilities, including enhancing the health care services setting by improving the level of patients’ attendance and minimizing the negative impact of waiting times. The first approach aims to change the behavior of patients by reminding them of their appointment by phone or providing motivating educational materials. The second approach assumes that it is difficult to change patient behaviors, and aims to minimize the no-show impact by enhancing the decision-making process regarding scheduling and resource allocation. An accurate estimate of patients’ attendance levels is likely to inform the evaluation and the adopted policy. An important aspect of improving management practices is the provision of actionable information. Examination of complex problems with fragmented data and multiple variables requires information systems to advance improved resource allocation and enhance the performance of health care facilities [12]. Therefore, researchers have applied mathematical models to determine optimal overbooking levels [6]. Overbooking has become a widely adopted strategy for managing revenue across various industries [1].

### 2.5 Scheduling and overbooking models

Patients’ characteristics, including medical condition, age, demographics, and environment, could be factors of the no-show behavior [13,14]. In response to a lack of effective queuing management and long wait times, researchers have proposed overbooking schemes, such as static overbooking, open access scheduling, and dynamic overbooking [[15], [16], [17], [18], [19]]. Most of these schemes use multi-objective optimization models to balance the revenue, time, and cost of appointment cancellations. In the static schemes, the number of potential patients is usually known, whereas dynamic schemes evolve based on appointment calls [9]. Parizi and Ghate [10] examined the possible benefits of classifying patients without making the customary assumption that they are all the same. Cancellations and no-shows are severe disruptions in appointment scheduling, and overbooking could be employed to overcome this challenge. The possible reason that researchers have largely refrained from constructing mathematical models and optimization algorithms for health care overbooking and managing no-shows is that such models would inevitably be large-scale, and precise solutions are computationally challenging.

Outpatient clinics record patients’ scheduling information, including cancellations and no-shows, which can be leveraged to predict the future probability of patient no-shows. Zheng et al. [20] categorized different appointment scheduling models into primary care and specialty clinics. Primary care schedules patients’ appointments with physicians upon patients’ request regarding specific health care concerns. This implies scheduling patients’ appointments within a fixed time interval. Specialty clinics schedule appointments for a specialty consultation based on referrals from primary care physicians for specific evaluations and treatments. Accordingly, scheduling appointments often conducted in batches within variable intervals.

Appointment scheduling system design considers three aspects of the block, the initial block, and the interval. The block indicates the number of patients who arrive early within an appointment period. The initial block indicates the number of patients who attended an initial appointment. Finally, the interval indicates the duration of the appointment interval that could be fixed or variable. Moreover, an appointment system can classify patients to ensure better use of projected service times; adjust walk-ins, urgent care, and emergency patients; and optimally control no-shows [21]. The three major types of appointment scheduling policies are unit-level, single batch, and periodic. A unit-level policy is implemented when an appointment request occurs once at random intervals, and is associated with the traditional first-call-first-scheduled model or open access scheduling models. A single batch policy is implemented when the appointments scheduled are not confirmed until all requests are received. Finally, periodic scheduling is applied when the appointments of a group of patients accumulate and are sent to the scheduling system all at once [20].

Agrawal et al. [7] argued that research on health care operational research has neglected patient preference when booking an appointment, as few studies have included this consideration when developing optimal scheduling policies. Moreover, the authors contended that the literature on appointment scheduling can be classified into intra-day and inter-day scheduling, wherein intra-day scheduling maximizes the use of physicians’ time and reduces patients’ waiting time on the same day, and inter-day scheduling books appointments by allocating excess patients to future days using a multi-day planning horizon. The slot scheduling method schedules patients’ consultation times and provides the optimal time interval for visiting the clinic to avoid queuing and long wait times. This method is more effective than scheduling patients at any time during a consultation session [6].

### 2.6 Overview of health care in Saudi Arabia

Saudi Arabia has one of the highest public health expenditures, raising concerns regarding the quality of health care system, and population growth of 3.2% per year establishes an alarming need [22]. The Saudi Ministry of Health [23] budget for 2021 was SR79,846,364 (almost equivalent to $21.3 billion) (https://www.moh.gov.sa/en/Ministry/About/Pages/Budget.aspx). Health care industry and providers in Saudi Arabia are expected to be among the top economic priorities, as the industry has high potential for growth. Alharbi [24] clarified that the previous government role was to follow a national health care model, in which the government is primarily responsible for the provision of health services and people received health care services free of charge.

Saudi Arabia launched its Vision 2030 in 2016, addressing three themes of fostering an ambitious nation, a vibrant society, and a prospering economy. An interim development plan entitled National Transformation Program (NTP) 2020 was established to formulate strategic objectives for all main economic sectors and required reforms, including the health care sector. NTP emphasized the necessity of improving health care service quality, the efficient use of facilities, and making services available to the population. In addition, NTP stresses the importance of private investment in the health care sector. Therefore, the 2030 Vision aims to liberalize the health care sector, increase the role of the private sector, and mitigate the pressure on the public sector [24].

In line with Vision 2030, the NTP and the Ministry of Health (MoH) have exhausted a budget close to $71 billion in the past five years, forecasting an annual growth rate of 12.3% in the health care sector by 2021. Additionally, the rising population (specifically those over 60 years of age) will encourage the adoption of mandatory health insurance for the nation [[25], [26], [27]]. Among the many goals of NTP programs, the most significant will contribute to increasing private health care expenditure by 35% by urging the expansion of licensed medical facilities from 40 to 100. This projected growth drive initiatives to decrease the proportion of obesity and smoking to a baseline incidence percentile range of 1% [[25], [26], [27]]. More importantly, the MoH is also implementing measures to improve the quality of health care services and increasing focused efforts on digitizing processes using the latest technological innovations. In summary, the current highest rated benefits to encourage investment in the region are structure, population, support for the private sector, and most importantly, generous budget allocations toward improving and satisfying the demand for health care basics and innovations.

Both the private sector and nongovernmental organizations can contribute to improving health care services in collaboration with the MoH. The NTP implies that the Saudi government will enhance the health care infrastructure, maintain safety, follow quality standards, and manage health facilities. According to the NTP, the government is required to build medical campuses across the country a total investment of $4.3 billion. In addition, there are 23 preexisting hospitals and 38 medical centers. The MoH is managing to accelerate the privatization of the health care sector, while the public sector will be engaged in the process of promoting health care services, advising people regarding the importance of primary healthcare, and reducing infectious diseases. This means that the role of the public sector is narrowing its role to supervising, planning, and monitoring the delivery of health care services. The private sector should ensure the introduction of high-quality health care services and reduce patient waiting times [28].

Congruent with previous initiatives, the health care sector transformation program under Vision 2030 was established in 2021 to restructure the sector over five years to be more effective and comprehensive, and to consider both individual and societal level effects. The program focuses on the main principle of value-based care, which is to maintain transparency and financial sustainability through the promotion of public health services and prevention of chronic diseases. This requires improving the access to services, consistent health care service quality, and maintaining equal geographical distribution by reaching distant areas and improving tele-health services [29].

The investment motivated the Kingdom of Saudi Arabia to actively seek and explore the growth of private sector involvement in developing a superior health care infrastructure through the introduction of the public–private partnership model. The program unlocks the value of the health care system resulting in a fast-track transformation plan to increase the private sector contribution to 35% by 2020. Additionally, research has showcased that national life expectancy is predicted to increase to 79 years of age for men and 82 years for females by 2050. This has also encouraged prosperity and potential levels of investment for educating the next generation of physicians or the provision of funding to continue operating and improving facilities to serve the expected 2050 population of 77.2 million people in the kingdom [30].

## 3 Method

This section presents the equations applied and introduces the proposed expected loss model. It is essential to identify the cost and capacity of each clinic to determine the maximum number of appointments that can be made. In general, medical center costs include salaries, operations costs, government fees, and space (rent). The following is used to calculate the capacity of each clinic:

(1) $$C_{a}^{C}=\sum_{n=1}^{N}\frac{V_{n}^{C}}{W_{n}^{c}}$$

where *C*_*a*_^C^ is defined as the overall capacity of clinic (***C***); *V*_*n*_^*C*^ is defined as the maximum number of consultations by physician ***n*** in clinic ***C***; and *W*_*n*_^*C*^ is defined as working hours per shift for physician ***n*** in clinic ***C***.

### 3.1 Probabilities of no-shows

The number of no-shows was calculated and detected over three months in each clinic. Based on this historical data, the probability mass function (PMF) and cumulative distribution function (CDF) of each clinic is calculated as follows:

If *S* is a discrete random variable (number of no-shows), then its range (*R*_*S*_) is a countable set; thus, we can list the elements in *R*_*S*_ as follows:

(2) $$R_{S}=\left\{s_{1},s_{2},s_{3},...\right\}$$

where *s*_*1*_*,s*_*2*_*,s*_*3*_, … represents possible values of the random variable *S*. The random variables denoted by capital letter *S* represent the numbers in the range of lowercase letters. For a discrete random variable S, we are interested in knowing the probabilities of *S* = *s*_*k*_. Note that here, the event *A* = *S = s*_*c*_} is defined as the set of outcomes (*s*) in sample space *Y* for which the corresponding value of *S* is equal to *s*_*k*_; in particular,

(3) $$A=\left\{y\in Y|S\left(y\right)=S_{c}\right\}$$

The probabilities of no-show events {*S = s*_*k*_} are formally shown by the PMF of *S*.

Let *S* be a number of no-shows variable with range *R*_*S*_ = { *s*_*1*_*,s*_*2*_*,s*_*3*_, … } (finite or countably infinite). The function

(4) $$P_{s}\left(sc\right)=P\left(S=sc\right),for\;c=1,2,3,...,$$

is the PMF of *S*.

The PMF is a probability measure eliciting probabilities of the possible values for a random no-shows variable. While the above notation is the standard method for the PMF of *S*, the subscript *S* indicates that this is the PMF of the random no-shows variable *S*. Thus, for example, *P*_*S*_(4) indicates the probability that the number of no-shows (*S*) = 4. To illustrate, if seven no-shows occurred four times over 30 days, then the probability of having seven no-shows on a given day would be *P*_*s*_(4)=(4/30).

### 3.2 Expected loss model

The loss model proposed in this study is expected to complement previously developed models by other researchers seeking to optimize health care clinics’ profits by minimizing patients’ no-show rates and waiting times. Similar relevant models are discussed next. Reid et al. [31] designed and tested a model to predict patient no-shows using passive information in a high-volume clinical environment. The model automated the prediction of no-shows and could benefit health care organizations by increasing service use and minimizing clinic overflow. Moreover, the authors assume that predictive overbooking is more impactful than fixed overbooking. Agrawal et al. [7] developed a more general appointment scheduling system than used in other research that assumed that physicians are the only resource required to schedule an appointment. In addition, the model assumed flexibility in allocating patients across various clinic locations and assumed that patients’ demands follow the normal distribution. This study also echoes Zheng et al. [20], who developed an overbooking scheduling model for multi-provider outpatient clinics, aiming to maximize profits. The authors proposed a comprehensive objective function considering important factors in clinics, including, revenue, staff overtime, and patients’ waiting times using a scheduling algorithm that investigated overbooking. Moreover, Muthuraman and Lawley [21] developed an overbooking model for outpatient clinics and a short scheduling policy to decrease the probability estimates of no-shows. In addition, the authors developed an objective function integrating measures of patient revenue, waiting time, and staff overtime to predict profits and the natural stopping criterion for the resulting scheduling policy. This study applies a different tool from that adopted by Al-Aomar and Awad [9], who developed a dynamic discrete clinic operations’ event simulation model to assess performance considering varied rates and patterns of patient no-show rates and tested many overbooking strategies to enhance the clinic’s efficiency and performance. The research significantly analyzed variations in treatment times and scheduling, the impact of reengaging patients, and walk-in patients. According to Fomundam and Herrmann [32] both queuing and simulation models have advantages, but queuing models require less data, are simpler, and produce more generic results than simulation models.

The main objective of the model below is to minimize no-show costs by identifying the optimal number of overbooking (*R*) for each clinic (*C*).

$$\mathrm{Min}.\;\sum_{c=1}^{C}\sum_{s=1}^{S}{P\left(s\right)}^{c}\times \left|\left({CF}^{c}+{OC}^{c}\right)*S^{c}-\left({CF}^{c}+{VC}^{c}\right)*R^{c}\right|$$

Subjectto:

(5) $$\left({CF}^{c}+{OC}^{c}\right)*S^{c}=\left({CF}^{c}+{VC}^{c}\right)*R^{c}$$

$${P\left(s\right)}^{c}=P\left(S=s^{c}\right),for\;c=1,2,3,\ldots \text{,}$$

$$S^{c}\geq 0$$

$$R^{c}\geq 0$$

where *R*^*C*^ is the number of overbooking reservations in clinic *C*. VC represents overbooking cost. C is the clinic number, where C = {1, 2, 3, …}. S^C^ is the number of no-shows in clinic C; CF^C^ represents the consultation fee in clinic C; OC^C^ is the operation cost of clinic C; SCF is the no-show cost factor; and P(s)^c^ represents the probabilities of *S* no-shows in clinic *C*. In the next section, the model is tested using clinical platform data to demonstrate the efficacy of the proposed models in minimizing expected no-show costs in outpatient clinics.

## 4 Case study

### 4.1 Introduction to the case study

The applied case study examines a local health care network based in Jeddah, Saudi Arabia that has been among the largest private health care providers in the past 60 years and was acquired by a major health care firm in the past five years. Since this acquisition, considerable changes have occurred, and the structural set of clinics has been shifted and organized to the following 14 clinics: Endocrinology/Diabetes, Obstetrics and Gynecology, Neurology, Psychiatry, Orthopedics, Oncology, Ophthalmology, General Surgery, Cardiology, Family Medicine, Dermatology, Internal Medicine, Pediatrics, and Pulmonology. The medical center is investigating the development of a plan to minimize losses and costs by determining the frequency of no-shows or missed appointments to strategize an intelligent overbooking scheduling solution. Each clinic has unique challenges, histories, and financial stressors. The following section presents an in-depth discussion of the case study, outlining the clinics’ structural backgrounds, along with the descriptive statistics regarding costs and no-show frequencies.

Table 1 presents the different clinical departments along with the number of working physicians and nurses, capacities, service fees, costs, and their shifts. All clinics are active and receive patients from Sunday to Thursday from 10:00AM to 10:00PM; however, actual staff working hours are from 9:00AM to 11:00PM for shift start and closing. The most high-pressure shift is the evening shift, and morning shifts are less crowded and have fewer intakes and working physicians on-call. Consultation fees are provided and differ per department and appointment status. Walk-in or unexpected appointments are charged at higher rates due to their rushed nature. Table 2 presents an outline of the costs for running the medical center.

**Table 1:** The medical center - general operational structure.

#	Clinic	Doctors	Nurses	Maximum Capacity Per Clinic	Consultation Fee	Operation Clinic Cost Per Patient (30 min)	Overbooking Cost Per Patient (30 min)
**1**	Endocrinology and Diabetes	3	6	36	SAR 300.00	SAR. 35	SAR 70.00
**2**	Obstetrics and Gynecology	4	8	48	SAR 400.00	SAR. 35	SAR 100.00
**3**	Neurology	3	6	36	SAR 500.00	SAR. 35	SAR 100.00
**4**	Psychiatry	4	8	48	SAR 500.00	SAR. 35	SAR 50.00
**5**	Orthopedics	3	6	36	SAR 500.00	SAR. 35	SAR 50.00
**6**	Oncology	3	6	36	SAR 400.00	SAR. 35	SAR 150.00
**7**	Ophthalmology	3	6	36	SAR 350.00	SAR. 35	SAR 100.00
**8**	General Surgery	6	12	72	SAR 500.00	SAR. 35	SAR 200.00
**9**	Cardiology	3	6	36	SAR 450.00	SAR. 35	SAR 100.00
**10**	Family Medicine	4	8	48	SAR 200.00	SAR. 35	SAR 140.00
**11**	Dermatology	5	10	60	SAR 550.00	SAR. 35	SAR 50.00
**12**	Internal Medicine	5	9	60	SAR 400.00	SAR. 35	SAR 70.00
**13**	Pediatrics	3	6	36	SAR 300.00	SAR. 35	SAR 150.00
**14**	Pulmonology	3	6	36	SAR 500.00	SAR. 35	SAR 250.00

**Table 2:** The medical center operation cost.

Total Cost Breakdown	
TC (Total Cost)/365 days	SAR. 14,883,519
TC (Total Cost)/hour	SAR. 70
TC (Total Cost)/30 min	SAR. 35
Utilities/Operating Cost (Per Year)	SAR. 150,000
Government Fees (Per Year)	SAR. 120,000
Space and Land (Per Year)	SAR. 500,000
Total salaries for doctors	SAR. 6,045,572
Total salaries for nurses	SAR. 5,556,747
Total salaries for administrative staff	SAR. 2,511,200

As illustrated in Table 2, cumulative annual sum exceeding 14 million riyals is required as a minimum to operate all 14 clinics, an approximation that is calculated by gathering and adding all the basic cost variables, such as salaries, utilities, government fees, and land costs. The cost is converted into time intervals, in the interest of the requirements of this study. Based on the queuing time frame outline (Fig. 1), a single appointment would take 30 min; therefore, the annual expenditure of SAR. 14,883,519 is reduced to 30 min, allocating SAR. 35 per appointment. The purpose of this time delineation is to understand exactly how much each patient’s 30-min appointment costs the clinic. This number adds great value for determining the optimal overbooking scheduling system for each clinic to calculate the expected loss for each booking strategy. After identifying the cost structure for each clinic and understanding the capacity framework, an overbooking loss table can be created to ascertain the best strategy.

### 4.2 Model implementation

For illustration, Clinic 1 is used as the primary example to demonstrate how it is possible to use frequency data to establish a best fit overbooking strategy through expected cost awareness. Clinic 1 is the Endocrinology and Diabetes department, and according to Table 1, it has three doctors and six nurses on staff, with a daily capacity of 36 patients and a regular consultation cost of SAR. 300. If all scheduled patients showed and additionally overbooked patients showed, the overtime cost would be SAR. 70 per patient. Table 3 presents the account of the number of no-shows, their frequency, and their probability. The objective of the table is to first detect the expected number of no-shows.

**Table 3:** Clinic 1 No-show probability data table.

Clinic 1: Endocrinology and Diabetes										
No-Shows (*S*)	1	2	3	4	5	6	7	8	9	10
Frequency (F_s_)	10	6	7	13	3	17	14	10	5	3
Probability p (*P(s)*^*c*^)	0.11	0.07	0.10	0.14	0.03	0.19	0.16	0.11	0.06	0.03
CDF	0.11	0.18	0.28	0.42	0.46	0.64	0.80	0.91	0.97	1.00

To devise an overbooking loss table, the first step is to outline the no-shows and the probability, with one to 10 overbooking appointments available, while also considering the cost in the outline by appointment. Zero is placed over a regressing horizontal slant to express no loss; for example, if a single no-show occurs and the clinic only overbooked for one person causing no actual monetary disadvantage.

The table is filled by considering the clinic operation cost per patient (SAR. 35) and the clinic cost if all reserved patients show in addition to the overbooked patients (SAR. 70).

Equation (5) is applied to identify the best fit strategy for overbooking. The probability of each no-show is multiplied by each reserved overbooked appointment and added with the entire column to determine the expected loss. This is applied to all possible ranges of numbers that could be overbooked, which is from one to 10 appointments overbooked in this scenario. The best fit for the overbooking strategy is the number that elicits the least acting expected loss. The lowest expected loss indicates that the clinic will use its resources wisely through an efficient overbooking scheduling system considering the least amount of cost for a scenario in which the schedule is full and overbooked patients may need to be re-referred. Table 4 indicates that overbooking six appointments is the best strategy to avoid wasting money, time, and clinic resources, as it only has an expected SCF of 74.28. This method of producing an overbooking loss table is applied in all clinics to separately determine the best strategy for each clinic, given the different variable values.

**Table 4:** Overbooking loss table for clinic 1.

		Reservations Overbooked									
*S*	*P(s)*^*1*^	1	2	3	4	5	6	7	8	9	10
1	0.11	0	35	70	105	140	175	210	245	280	315
2	0.07	35	0	35	70	105	140	175	210	245	280
3	0.08	70	35	0	35	70	105	140	175	210	245
4	0.15	105	70	35	0	35	70	105	140	175	210
5	0.03	140	105	70	35	0	35	70	105	140	175
6	0.19	175	140	105	70	35	0	35	70	105	140
7	0.16	210	175	140	105	70	35	0	35	70	105
8	0.11	245	210	175	140	105	70	35	0	35	70
9	0.06	280	245	210	175	140	105	70	35	0	35
10	0.03	315	280	245	210	175	140	105	70	35	0
**SCF**		148.17	120.94	98.39	82.83	77.39	**74.28**	84.39	105.39	134.17	166.83

## 5 Results and discussion

**Fig. 2:**
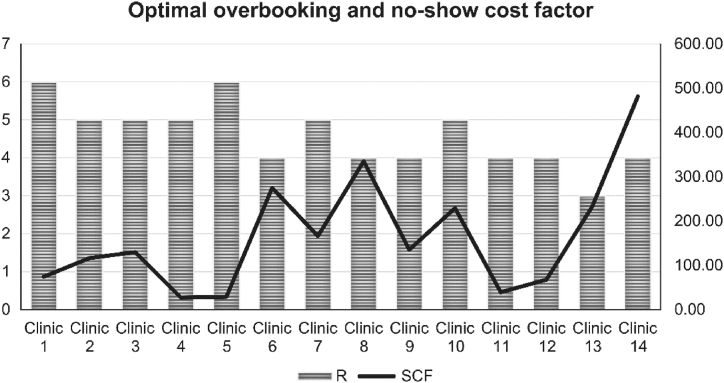
Clinic overbooking strategy expected loss output summary result.

After implementing the overbooking loss table and identifying the expected loss for each clinic individually, the results for the best optimal overbooking solution per clinic are presented in a bar graph on Fig. 2, showing the clinic number on the x axis and the best fit of the number of patients to overbook on the left y axis, and the expected return from each patient that is overbooked on the right y axis. The graph illustrates that the best choice is to pick the least regressed number for each clinic. The strategy considers variables such as frequency, each clinic’s patient holding capacity, and associated cost variables. The results presented in Fig. 2 indicate that it would be best for clinic 1 to overbook for six patients. This cost-saving overbooking plan could save each clinic a considerable sum amount, rather than randomly overbooking without any cost presumption or empirical investigation. The same strategy applies to all other clinics. Table 5 summarizes the results for the 14 clinics. For example, the optimal overbooking number (R) for clinic 7 is five appointments.

**Table 5:** Clinic overbooking strategy expected loss output summary results.

Expected Loss for Reservation Overbooked										
Appointments Overbooked	1	2	3	4	5	6	7	8	9	10
Clinic 1	148.17	120.94	98.39	82.83	77.39	**74.28**	84.39	105.39	134.17	166.83
Clinic 2	250.41	200.33	158.77	127.87	**116.15**	130.00	160.90	208.85	271.72	334.59
Clinic 3	239.69	195.00	162.50	142.19	**130.00**	134.06	162.50	219.38	280.31	345.31
Clinic 4	53.91	44.13	35.65	29.35	**27.39**	31.52	40.43	53.26	66.96	81.09
Clinic 5	68.10	54.30	42.30	33.90	30.30	**29.10**	33.90	40.50	53.10	66.90
Clinic 6	445.98	359.02	311.34	**274.88**	283.29	319.76	367.44	420.73	490.85	589.02
Clinic 7	305.74	245.56	199.81	173.33	**166.11**	168.52	175.74	192.59	223.89	279.26
Clinic 8	475.30	384.18	342.31	**334.93**	376.79	458.06	578.73	714.18	859.48	1009.70
Clinic 9	244.61	193.29	159.08	**135.13**	145.39	159.08	183.03	227.50	278.82	340.39
Clinic 10	439.25	355.25	299.25	260.75	**229.25**	239.75	278.25	334.25	411.25	505.75
Clinic 11	58.31	49.65	42.25	**39.08**	39.30	41.62	44.79	51.34	62.96	76.69
Clinic 12	118.05	87.80	71.78	**67.63**	78.90	92.54	110.93	136.44	164.32	196.95
Clinic 13	304.27	246.77	**232.40**	246.77	285.10	352.19	428.85	519.90	620.52	730.73
Clinic 14	733.80	602.93	528.15	**481.41**	490.76	565.54	668.37	789.89	986.20	1201.20

Overall, if the clinics implemented this strategy, they would incur no more than SAR. 2, 408 expected loss from overbooking as opposed to the exponentially increasing amount of SAR. 10,000 that is wasted annually on scheduling errors. The results clearly signify the expected loss difference through the line cutting through the bar graph showing variations in loss.

## 6 Conclusion

The objective of this study was to deploy a case study in the health care industry to examine the factors that impact the disruptions in efficient scheduling systems. The results demonstrated that constructing a cost-saving scheduling system requires the selection of a set of trending variables, including frequency, probability, capacity, and clinic cost expenditure. This sets the foundation for constructing the overbooking loss table and implementing the set equation to determine the cumulative expected loss and the fittest scheduling protocol for implementation. To benefit from implementing the model, whether in healthcare, airline, hotel, or other relevant industries, there historical data must be available to construct an appropriate model and structure for implementation. This tool is recommended for use as a decision-making aid and to identify malfunctioning cost variables. The expected loss table is a tool to inform and support change based on a comprehensive review of costs.

Three limitations are noted in the current study. First, the model constructed is tested considering a case study of a hospital network in Saudi Arabia, which limits the application of the model and lacks generalizability to similar cases. Second, the model is generic and does not consider patients’ gender, age, health condition, or geographic location; hence, it lacks controls or comparable groups. Third, the model was not empirically validated by the hospital management to monitor its deficiencies. Future investigations are expected to use this model and integrate outpatients’ demographic, geographic, seasonality, and health conditions. In addition, future research should consider the balance between no-shows and panel size.

## Author contribution statement

Abdulaziz Almaktoom; Conceived and designed the experiments; Performed the experiments; Analyzed and interpreted the data; Contributed reagents, materials, analysis tools or data; Wrote the paper.

## Data availability statement

No data was used for the research described in the article.

## Declaration of competing interest

The authors declare that they have no known competing financial interests or personal relationships that could have appeared to influence the work reported in this paper.
